# An alien parasite affects local fauna—Confirmation of *Sinergasilus major* (Copepoda: Ergasilidae) switching hosts and infecting native *Silurus glanis* (Actinopterygii: Siluridae) in Hungary

**DOI:** 10.1016/j.ijppaw.2021.04.011

**Published:** 2021-04-28

**Authors:** Quinton Marco Dos Santos, Annemariè Avenant-Oldewage, Wojciech Piasecki, Kálmán Molnár, Boglárka Sellyei, Csaba Székely

**Affiliations:** a Department of Zoology, University of Johannesburg, Auckland Park, Johannesburg, South Africa; b Institute of Marine and Environmental Studies, University of Szczecin, Poland; c Fish Pathology and Parasitology Research Team, Institute for Veterinary Medical Research, Centre for Agricultural Research, Budapest, Hungary

**Keywords:** Copepoda, Biological invasion, Wels catfish, Parasites, Danube river basin, Pathology

## Abstract

In 2016, an intense copepod infection was recorded from a reservoir in proximity to the Danube River in Hungary from visibly emaciated wels catfish, *Silurus glanis*. The parasite-induced pathology was described but parasite identity was not conclusive. Additional sample collections in 2017 and 2018 allowed for identification using both light and scanning electron microscopy, alongside genetic characterisation. The copepods were confirmed to be ergasilids, *Sinergasilus major*, distinctly different from any previous infection on silurids in Europe. This is the first record of this parasite from Hungary and the first host record from wels catfish.

## Highlights

• First record of *Sinergasilus major* from wels catfish (*Silurus glanis*).
• First record of *S. major* from Hungary.
• Light and scanning eletron microscopy support identification of *S. major.*
• 100% similarity of the 18S rDNA provides significant confidence to the identification of the copepods as *S. major.*

## Graphical abstract

**Figure:**
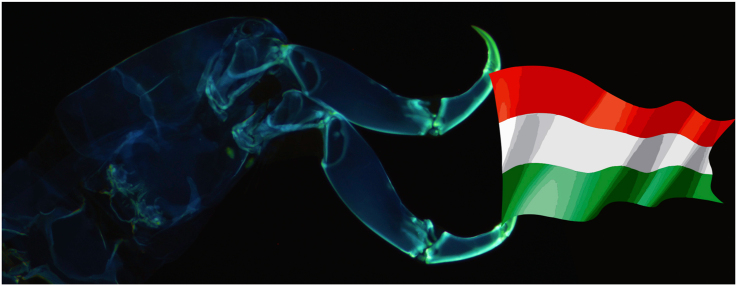

The genus *Sinergasilus* was erected by Yin (1949) with the description of *Sinergasilus lieni* Yin (1949) from *Hypophthalmichthys nobilis* (Richardson, 1845) and *Hypophthalmichthys molitrix* (Valenciennes, 1844) and *Sinergasilus yuii* Yin (1949) from *Ctenopharyngodon idella* (Valenciennes, 1844) in China. Thereafter, Yin (1956) reassigned *Pseudergasilus polycolpus* Markewitsch (1940), *Pseudergasilus major* Markewitsch (1940), and *Pseudergasilus undulatus* Markewitsch (1940) to *Sinergasilus*, synonymising *P. polycolpus* with *S. lieni*, and *S. yuii* with the new combination *Sinergasilus major* (Markewitsch, 1940)*.* As such, only three species of the genus are currently accepted, although there appears to be some confusion about which synonym between *S. lieni* and *S. polycolpus* is valid (*S. lieni* will be used here following Yin (1956) and Bykhovskaya-Pavlovskaya et al. (1962, 1964)). *Sinergasilus lieni* has spread from its original range (freshwater systems in China and the Amur River basin) and has been reported from Japan (Nitta and Nagasawa, 2020), across Russia (Mirzoeva, 1972; Musselius, 1969), fish farms in Macedonia (Dimovska and Stojanovski, 2015), ponds in Hungary (Molnár and Szé) and the Danube River (Cakic et al., 2004; Djikanović et al., 2018), but seems to be specific to the *Hypophthalmichthys* spp. as it has not been recorded from other hosts and has, therefore, presumably, been co-introduced to the new localities. Similarly, *S. undulatus* has also mostly been reported from hosts of the same genus from which it was described, *Carassius* sp., but also from common carp, *Cyprinus carpio* Linnaeus, 1758, but mainly in the Amur River basin (Bykhovskaya-Pavlovskaya et al., 1962, 1964; Markewitsch, 1940).

*Sinergasilus major* has not only been recorded from at least seven host species other than the type host *C. idella*, but their hosts are all from different genera and belong to four distinct families: four Cyprinidae (*Elopichthys bambusa* (Richardson, 1845), *Scardinius erythrophthalmus* (Linnaeus, 1758), *Mylopharyngodon piceus* (Richardson, 1846), *Squaliobarbus curriculus* (Richardson, 1846)), one Siluridae (*Silurus asotus* Linnaeus, 1758), one Bagridae (*Tachysurus fulvidraco* (Richardson, 1846)) and one Sinipercidae (*Siniperca chuatsi* (Basilewsky, 1855)) (Bykhovskaya-Pavlovskaya et al., 1962, 1964). Even though *S. major* appears to have a wider host specificity than the other two *Sinergasilus* species, it has mostly been recorded in the Amur River basin. However, since 1963 *S. major* has been recorded in other Eurasian systems outside of its native range, but always from the type host *C. idella*, with the copepod noted to be strictly specific to this host with which it was co-imported (Bauer et al., 1973). To date, infections of *Sinergasilus* spp. have not been recorded from indigenous fishes outside of the areas from which they were described.

Nevertheless, that was the case in 2016, when intense copepod infections were recoded from wels catfish from a reservoir in Pannonia, Hungary. At that time, the parasite-induced pathology of severely emaciated fish was described (Molnár et al., 2018). Unfortunately, the parasites were misidentified as *Lamproglena* sp*.* and prepared for pathological sections, resulting in limited samples for taxonomic identification. During 2017 and 2018, additional copepod samples were collected from catfish from the same reservoir, enabling more comprehensive identification.

Wels catfish were collected from a reservoir in Pannonia, Hungary (46°24′52.3″N, 17°59′31.3″E), transported alive to the laboratory in oxygenated water, held in concrete basins in flowing water, sedated with 20 ppm clove oil (Javahery et al., 2012), killed by a blow to the head, and the copepods were removed from the gills. Parasites were stored in either 70 or 96% ethanol for microscopic and molecular analyses, respectively. To determine the identity of the ergasilids, the specimens were studied using microscopy, both light (LM) and scanning electron (SEM), and DNA barcoding approaches. For LM, specimens were cleared in lactic acid and studied using a temporary mount. For SEM, whole specimens (6) were prepared by dehydration through a graded ethanol series, followed by a graded series of hexamethyldisilazane (Merck, Darmstadt, Germany) (Nation, 1983; Dos Santos et al., 2015). Specimens were then dried in a Sanpla dry keeper desiccator cabinet (Kita-Ku, Osaka, Japan), coated with gold using an Emscope SC500 sputter coater (Quorum Technologies, Newhaven, UK), and studied at 5–6 kV using a Vega 3 LMH scanning electron microscope (Tescan, Brno, Czech Republic). For molecular analyses, genomic DNA was extracted from ethanol fixed specimens (8), with exoskeletons retained and studied using LM as described. The genetic characterisation was based on two fragments of rDNA, 18S and 28S, using the primers and reaction conditions of Song et al. (2008). Amplicons were sequenced in both directions, merged, primers removed, and analysed using BLAST. Sequence data were then aligned to the closest matches, pairwise distances estimated by both uncorrected *p*-distance with 1000 bootstrap replicate variance estimation and the number of base pair differences using MEGA7, and evolutionary history assessed using both maximum likelihood (ML) and Bayesian inference (BI) methods in MEGA7 and BEAST v2.5.0 respectively. Representative sequence data were deposited to GenBank (18S - **XXXXXXXX**; 28S - **XXXXXXXX**).

Upon further inspection, the copepods were identified as Ergasilidae von Nordmann, 1832, and using the keys of Bykhovskaya-Pavlovskaya et al. (1962, 1964) and Boxshall and Halsey (2004) for genera of this group, the obtained morphological results indicated that the specimens in question represent *Sinergasilus* Yin, 1949. This is based on: the absence of a separation of the cephalosome and pedigerous somites; pedigerous somites almost equal in width; prosome not tapering posteriorly; body cyclopiform; external segmentation; stylets on cephalic shield absent; antennae not interlocking, with a single claw free from cuticular membrane or distal barb; second segment of antenna without teeth; first swimming leg without modified endopod, modified spine on exopod, or process on basis; fourth leg present and biramous; clear separation of pedigerous somites and abdomen; no digitiform processes at caudal ramus. Following this, using the key to *Sinergasilus* species by Bykhovskaya-Pavlovskaya et al. (1962, 1964), the specimens were further identified as *S. major* based on the more elongated body; smaller genital somite compared to the first abdominal somite; and the fourth pedigerous somite which does not cover the fifth pedigerous and genital somites (Fig. 1A). This identification is supported by the close similarity of the SEM observations to those of Huang et al. (1992) and Zhu et al. (2010) in that the integumental pores and tactile setules on the rostral plate (Fig. 1 B), and the thoracic plates with pectinate denticles (Fig. 1C) are nearly identical to that of *S. major*, clearly distinct from both *S. lieni* and *S. undulatus.*

**Fig. 1:**
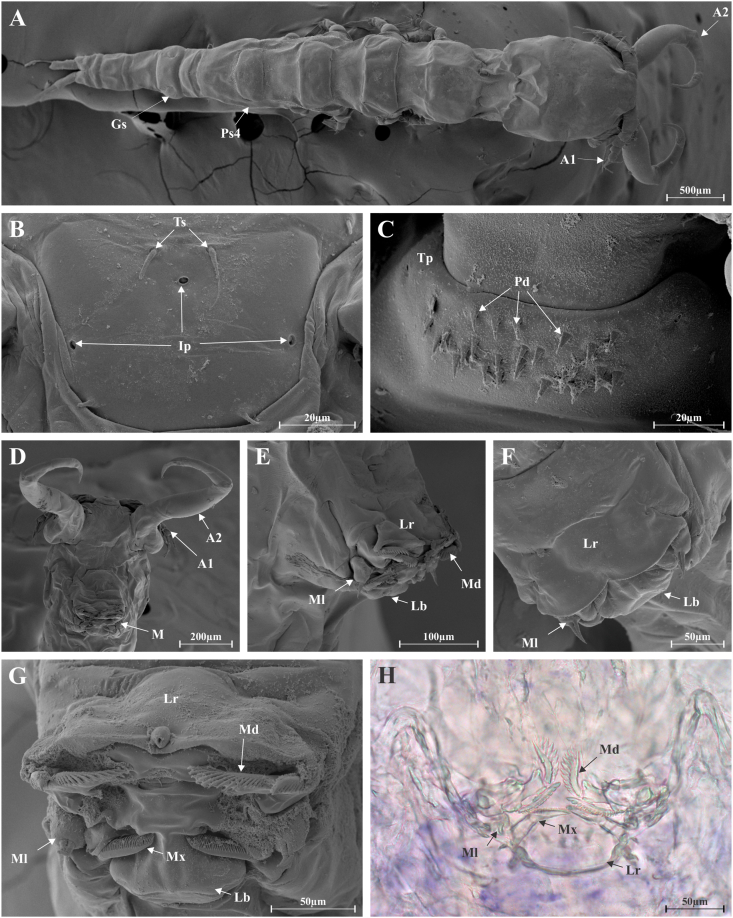
Micrographs using light microscopy (LM) and scanning electron microscopy (SEM) of *Sinergasilus major*; (A) Total body (SEM), (B) rostral plate with integumental pores and tactile setules (SEM), (C) thoracic plate with pectinate denticles (SEM), (D) ventral aspect of cephalon (SEM), (E) everted mouth (SEM), (F) inverted mouth (SEM), (G) ventral view of mouth parts (SEM), (H) mouth parts (LM). A1 – antennule 1, A2 – antenna, Ip – integumental pore, Gs – genital somite, Lb – labium, Lr – labrum, M – mouth, Md – mandible, Ml – maxillule, Mx – maxilla, Ps4 – pedigerous somite 4, Pd – pectinate denticles, Tp – thoracic plate, Ts – tactile setules.

Regarding the genetic characterisation, both rDNA fragments confirm the identification of the current material as *Sinergasilus*. For the 18S rDNA, all samples produced a single haplotype (1405 bp) which was identical to that of *S. major* collected from *E. bambusa* in the Danjiangkou Reservoir, China, only differing from *S. major* collected from *C. idella* and *S. curriculus* by 0.07% (1 bp), confirming the morphological identification (see Table 1). This also matches the observed intra- and interspecific ranges for the genus of 0–0.15% and 0.22–0.58% respectively. Evolutionary history analyses of 18S rDNA (Fig. 2) indicate a monophyletic grouping of the generated haplotype with other data for *S. major*, which is sister to a clade of the remaining *Sinergasilus*. Similar to what was observed by Song et al. (2008) the three *Sinergasilus* species form a monophyletic clade, but this group is within the ingroup of *Ergasilus*. Interestingly, the 28S rDNA (single haplotype, 650 bp) produced was not similar to available sequence data for *S. major*, even though the available data was produced from the same parasite and the same host, locality and study as the aforementioned 18S rDNA data by Song et al. (2008). It is possible that the available 28S rDNA sequence for *S. major* contains some errors as it is substantially distant from the other *Sinergasilus* spp. then they are to one another. Additionally, comparing the sequence in alignment to the other ergasilids, there is variation at otherwise conserved sites, indicating possible previous sequencing errors. As such, the distance and evolutionary history analyses based on 28S rDNA did not provide much valuable information at that time. This needs further investigation and the addition of more data, especially as all the ergasilid data for the markers used were from a single study. However, the 100% similarity of the 18S rDNA still provides significant confidence to the identification of the copepods as *S. major*.

**Table 1:**
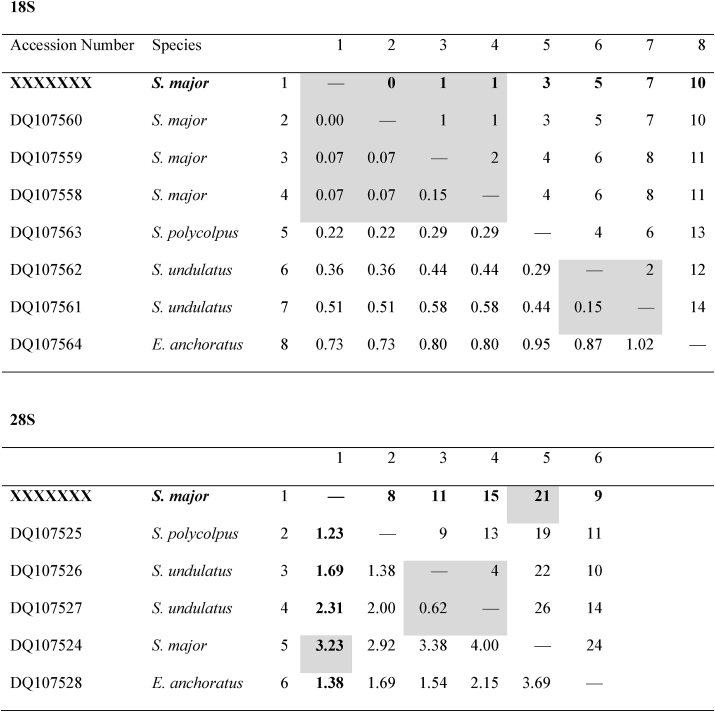
Estimates of evolutionary divergence between *Sinergasilus* spp. and *Ergasilus anchoratus* using both 18S and 28S rDNA. Sequence distances calculated using both *p*-distance (%) and number of base pair differences indicated below and above the diagonal, respectively. Shaded cells indicate intraspecific variation.

**Fig. 2:**
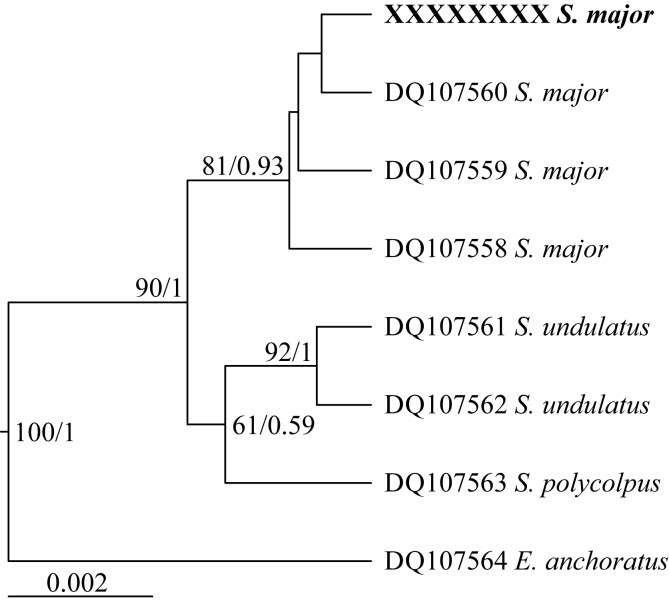
Evolutionary history of *Sinergasilus* based on Bayesian Inference (BI) analysis of 18S rDNA with *Ergasilus anchoratus*Markewitsch, 1940 designated as outgroup. Support for both maximum likelihood (ML, 1000 bootstrap replicates) and BI (10 million MCMC) indicated at nodes (ML/BI), only nodes with more than 50% support annotated.

The identical sequences from the current material and that from *E. bambusa* in China may indicate a possible fish species on which *S. glanis* was translocated to the Danube. But, to our knowledge there is no record of this fish species in the Danube River basin, making the introduction of *S. major* more likely via the type host *C. idella*. This cyprinid is known to occur in the system, with records of *S. major* infecting *C. idella* being noted in Europe since 1955 (Bauer and Babaev, 1964; Musselius, 1969; Bauer et al., 1973; Yevtushenko, 2020) and even in the Danube Delta (Angelescu, 1981). All three *Sinergasilus* spp. have been described from cyprinid hosts. This discovery of *S. major* on wels catfish is not surprising as *Sinergasilus major* has been reported from eight genera and four families, including the silurid congener to *S. glanis*, the Amur catfish *S. asotus*. Furthermore, the record of *Lamproglena pulchella* from wels catfish by Kurbanova et al. (2002), may have been a misidentification and in fact be the first record of *S. major* from *S. glanis*. The fact the catfish examined were emaciated is concerning as this may have been caused by invasive *S. major*.

As mentioned, the pathology of copepods studied here has been described in an earlier publication (Molnár et al., 2018) where they were misidentified as *Lamproglena* sp. The catfish, infected with hundreds of copepods (200–450 per fish), were also visually emaciated. Like L. *pulchella*, they attached to the distal part of the gill filaments, leading to hypotrophy of epithelial tissue (Molnár et al., 2018). Interestingly, no egg sacs were observed, as is the case in the present study. As the pathology described by Molnár et al. (2018) was linked to a misidentified copepod, re-evaluation of the effect on the host is paramount. Considering the morphology of mouthparts of the current specimens (see Fig. 1D–H), it is clear that they differ from those of *Lamproglena* and the pathology should be related to that caused by *S. lieni* previously reported (Molnár and Szé). Continuous monitoring of the condition of the native wels catfish is also required as they were co-infected with *Proteocephalus osculatus* Goeze, 1782 (Cestoda, Proteocephalidae) which also may have attributed to their poor condition.
